# Errata

**Published:** 1856-08

**Authors:** 


### ERRATA.

In the May number of this Journal, page 518, "Article 3d," for "Abnormal Placenta, by Dr. J. R. Johnson, Lebanon, Alabama," read Abnormal Placenta, by Dr. H. H. Johnson, Fair Play, Alabama.

In July number, page 691, article, "New Orleans School of Medicine," for "Dr. Erasmus and Dr. Fenner," read Dr. Erasmus D. Fenner.

